# Relation of the left ventricular geometric patten to coronary artery disease in hypertensive patients using a 320-detector-row CT scanner

**DOI:** 10.1186/s43044-023-00360-7

**Published:** 2023-04-26

**Authors:** Ahmed Shehata Mohamed Ismail, Mohamed Ahmed Aouf, Reda Hussein Diab, Yasser Kamel Baghdady

**Affiliations:** Cairo University, Cairo, Egypt

**Keywords:** Left ventricular hypertrophy, CT, LV mass, LV geometric pattern

## Abstract

**Background:**

It was estimated that about 1.3 billion people were diagnosed to be hypertensive in 2015. All countries consistently show this high prevalence. Ischemic heart disease stands as the most common cause of systolic blood pressure-related deaths per year. Left ventricular hypertrophy determined by echocardiography can predict cardiovascular morbidity and mortality. The question of whether the LV geometric pattern has an additional prognostic value is still not clearly answered. Currently, coronary computed tomography is widely used in clinical practice with a great capability of simultaneous evaluation of the LV mass and the coronary arterial tree. Our study aims to examine the relationship between LV mass and geometry and coronary artery disease using an ECG-gated 320-detector- row CT scanner.

**Results:**

Two hundred ninety-eight hypertensive Egyptian individuals were enrolled in our study, the mean age was 57.5 ± 10.5, and males comprised 76.5% of the study population. The mean LV mass and LV mass index were 193 ± 60 gm and 95.2 ± 27.5 g/m^2^ respectively. One-fifth of the patient had CAD luminal stenosis ≥ 50%. Normal LV geometric pattern was observed in about 37% of the study population. About one-third of the patients showed concentric remodeling. Patients with increased LV mass index represented one-third of the study population with a greater percentage of the concentric hypertrophy pattern than the eccentric hypertrophy pattern. Patients with high CAD-RADS showed statistically significant higher LV mass, LV mass index, and septal wall thickness. Patients with high CAD-RADS showed a greater percentage of concentric and eccentric hypertrophy. The LV geometric pattern was the only independent predictor of the high CAD-RADS. The LV geometric patterns associated with high RADS ordered from the highest to the lowest, were concentric LVH, Eccentric LV, and concentric remodeling.

**Conclusions:**

LV geometric pattern is the only independent predictor of high CAD-RADS after adjustment for LV mass index and septal wall thickness. Among abnormal LV geometric patterns, concentric hypertrophy stands as the most important predictor of high CAD-RADS.

## Background

It was estimated that about 1.3 billion people were diagnosed to be hypertensive based on office blood pressure in 2015. All countries consistently show this high prevalence irrespective of their economic status [1, 2].

The disability-adjusted life years owing to hypertension have increased by 40% since 1990. Ischemic heart disease stands as the most common cause of systolic blood pressure-related deaths per year [3].

Chest X-ray and ECG are insensitive techniques for the detection of left ventricular hypertrophy. On the other hand, LVH determined by echocardiography has a well-established prognostic value in predicting cardiovascular morbidity and mortality [4–11].

The geometric pattern of hypertrophy of the left ventricle associated with arterial hypertension is identified by calculating the LV mass and the relative wall thickness. The question of whether the LV geometric pattern has an additional prognostic value is still not clearly answered [10, 12–14].

Coronary computed tomography is widely used in clinical practice for the non-invasive evaluation of the coronary arterial tree. The three-dimensional capability of the ECG-gated CT study allowed for the simultaneous, accurate evaluation of the LV volumes and mass. Its accuracy has been validated against cardiac magnetic resonance imaging –the gold standard for LV volume and structure assessment– in previous studies [15–19].

Our study aims to examine the relationship between LV mass and geometry and coronary artery disease using an ECG-gated 320-detector- row CT scanner that can simultaneously evaluate the coronary arterial tree and the LV chamber size and mass.

## Methods

### Study design and population

This is a prospective, non-randomized, cross-sectional, observational study that enrolled 298 hypertensive patients scheduled for MSCT coronary angiography for suspected coronary artery disease.

### Consent for publication

All patients gave written informed consent to publish the data contained within this study.

### Inclusion criteria

Hypertensive Individuals aged over 30 years who were scheduled for MSCT coronary angiography. Hypertension is defined as office BP measurements of SBP ≥ 140 mmHg and/or diastolic BP (DBP) ≥ 90 mmHg [20].

### Exclusion criteria

1. Diabetes mellitus (DM), dyslipidemia, or smoking.
2. Valvular heart disease.
3. Cardiomyopathy.
4. Previous coronary artery bypass (CABG) or percutaneous coronary intervention (PCI).
5. Individuals who refuse to participate in this study.

### Study design

1. Clinical assessment: All individuals were subjected to focused medical history and clinical examination with emphasis on the following:
  - Demographic characteristics and anthropometric measurements:
  Age and genderb-Cardiovascular risk factors: DM, dyslipidemia, and smoking.
  Height measurement using the standing height scaleb-Cardiovascular risk factors: DM, dyslipidemia, and smoking.
  Body weight and body mass index (BMI). The latter was calculated using the following formula: BMI = body weight in kg / height in m^2^). Adults were classified according to BMI (table adapted from WHO Consultation on Obesity): Underweight < 18.5 Normal range 18.5–24.9 Overweight 25–29.9 Obese class I 30–34.9 Obese class II 35–39.9 Obese class III ≥ 40 [21].b-Cardiovascular risk factors: DM, dyslipidemia, and smoking.
  Body surface area (BSA): This was calculated using Du Bois formula: BSA = 0.007184 × weight in kg^0.425^ × height in cm^0.725^.b-Cardiovascular risk factors: DM, dyslipidemia, and smoking.
2. Multi-slice CT Angiogram (MSCT) Study Protocol:

A retrospective, ECG-gated multi-slice CT coronary angiography was done using a 320-slice, Toshiba Aquilion one machine (Kern: FC43, spacing 0.25 mm, FOV 240 mm, thickness 0.5 mm, 120 kV, 450 mA). After acquiring the non-contrast enhanced sequence, a high flow rate injection of ''A patient-weight tailored contrast dose ''average of 75 ml of non-ionic contrast (5.3 ml/sec.) via an anti-cubital vein was done, followed by the acquisition of ultra-thin cardiac sections to evaluate the coronary arteries. The study was evaluated at the systolic and diastolic phases (75% and 40% of the cardiac cycle, respectively). Selective reconstruction of the poorly visualized segments of the coronary arteries at different phases of the cardiac cycle was done. Coronary artery disease reporting and data system (CAD- RADS) was used to standardize our reports [22].

Additional images to evaluate the LV diameters, LV wall thickness, and LV volume was reconstructed during diastole with increments of 5% from 75 to 90% of the RR interval. Data were transferred to a workstation with commercial software (Vitrea FX, version 6.1, Vital Images, Minnetonka, MN, USA) for analysis. Multi-planar reformation (MPR) images of the LV cardiac chamber (including short-axis, 4-chamber, and 2-chamber views) were automatically generated.

**Fig. 1:**
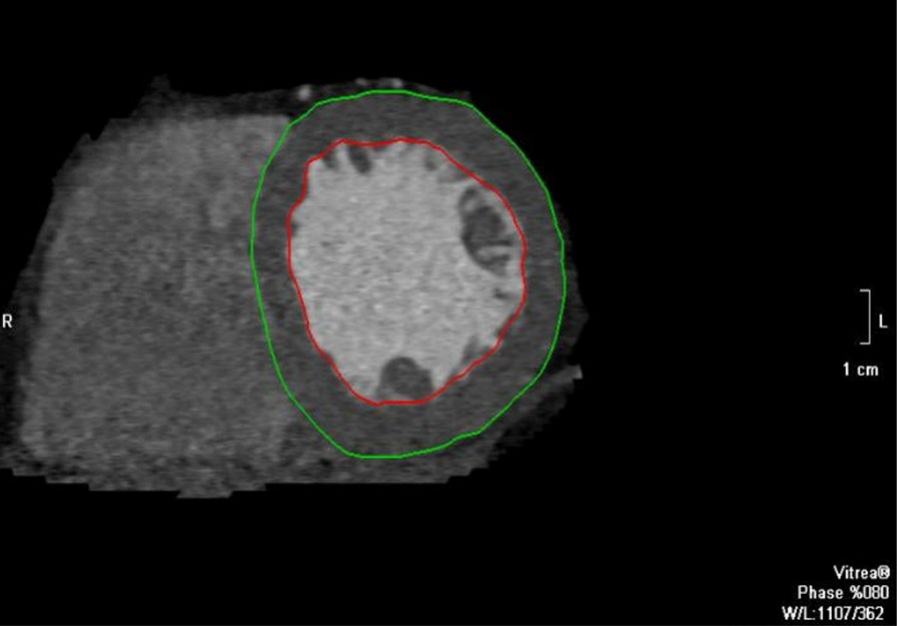
Assessment of the LV mass by automatic contouring of the endocardial and epicardial borders with slight manual correction, taken at the end-diastole (80% of the cardiac cycle)

The LV volumes were calculated by automatic detection of the LV epicardial and endocardial borders by the software without the inclusion of the papillary muscles (Fig. 1). Both borders could be corrected by the reader (expert cardiologist) if necessary. The end-diastolic phase was defined as the largest LV volume. These estimates were used to calculate the LV myocardial mass (g) ([epicardial volume—endocardial volume] × 1.04 g/mL), where 1.04 is the myocardial density. The LV mass was normalized using BSA (m^2^). The cutoffs of 115 g/m^2^ for men and 95 g/m^2^ for women were used to define the presence of LVH [23, 24].

**Fig. 2:**
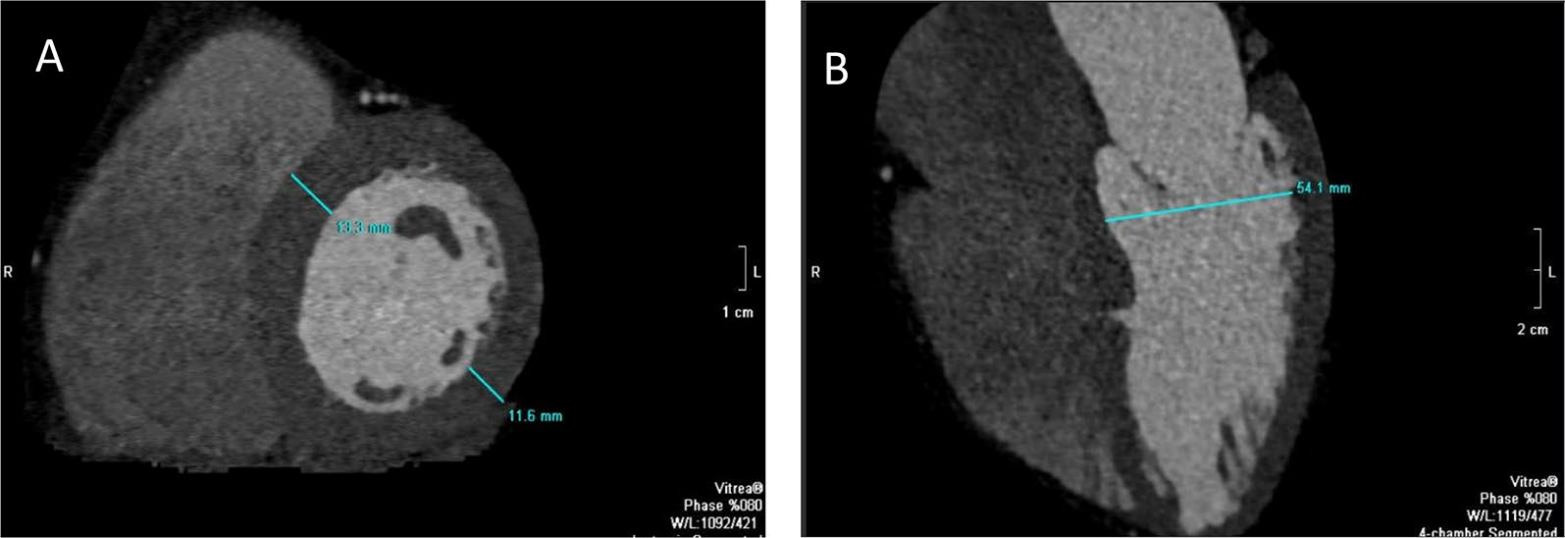
Measurement of the LV wall thickness (both the septum and posterior wall) in the short axis at the level of the chordea (**A**) and the left ventricle end-diastolic inner diameter in the four-chamber view (**B**)

The LV end-diastolic inner dimension (LVID, cm) on the 4-chamber multi-planar reformatted images (MPR), LV wall thickness; both the septum (septal wall thickness [SWT], cm) and the posterior wall (PWT, cm) on the short-axis MPR at the chorda level were measured (Fig. 2). The relative wall thickness was calculated as (RWT [cm] = 2 × PWT / LVID). A value of > 42% was defined as abnormal [23, 24].

The patients were classified into four groups of geometric patterns based on the values of LV mass index and RWT: normal (normal LV mass and normal RWT), concentric remodeling (normal LV mass and increased RWT), eccentric LVH (increased LV mass and normal RWT), and concentric LVH (increased LV mass and RWT) [25]. To assess the inter-observer variability, the first 20 subjects were analyzed by two experienced readers (two cardiologists who are experts in cardiac imaging).

### Statistical methodology

After data collection, analysis was done using Statistical Package of Social Science (SPSS) version 19. Categorical data were displayed as numbers and percentages and continuous data were displayed as means ± SD (for normally distributed data) or median and range (for abnormally distributed data). Comparison analysis was conducted using student’s t-test (for data that were normally distributed) or a Mann–Whitney test (for data that were not normally distributed) and a Pearson Chi-square test (for categorical variables). Multiple linear regression analysis was used to predict significant CAD. A two-tailed *P*-value < 0.05 was considered significant.

### Reproducibility

CT data (CT coronary angiography and LV measurements) of the first twenty patients were repeated by the same operator one week later to test for intra-observer variability, and the intra-observer correlation coefficient ranged from 0.88 to 0.98. The CT data of the same twenty patients was measured by another operator to test for inter-observer variability and the intra-observer correlation coefficient ranged from 0.89 to 0.97.

## Results

This non-randomized, observational, cross-sectional study was conducted in Cairo, Egypt from November 2019 to November 2021. This study enrolled 298 Egyptian patients who underwent CT coronary angiography for suspected CAD.

### Demographics and baseline clinical characteristics of the study population

Two hundred and ninety-eight Egyptian individuals were enrolled in our study. The mean age was 57.5 ± 10.5, males comprised 76.5% of the study population, the mean weight and height were 87.6 ± 15.7 kg and 171.1 ± 8.0cm respectively, the mean BMI was 29.6 ± 5.1 cm^2^/kg and the mean BSA was 20 ± 0.2 m^2^. About two-thirds of the study population were overweight or mildly obese. These data are presented in tables 1 and 2.

**Table 1:** Baseline clinical data of the study population

Variables	Median (range)
Age (years)	57.3 ± 10.5
Gender, male	228 (76.5%)
Height (cm)	171.1 ± 8.0
Weight (kg)	87.6 ± 15.7
BMI (cm^2^/kg)	29.6 ± 5.1
BSA(m^2^)	20 ± 0.2

**Table 2:** Obesity distribution among the study population

BMI category	Number	Percent (%)
Underweight (< 18.5)	3	1
Normal (18.5–24.9)	51	17.1
Overweight (25–29.9)	102	34.9
Obesity Class I (30–34.9)	100	34.2
Obesity Class II (35–39.9)	30	10
Obesity Class III (≥ 40)	12	4

The mean LV mass and mean LV mass index were 193 ± 60 gm and 95.2 ± 27.5 g/m^2^ respectively. The CAD RADS score shows a median of 2. Only 20% of the patient had CAD luminal stenosis ≥ 50%. Normal LV geometric pattern was observed in about 37% of the study population. About one-third of the patients showed concentric remodeling. Patients with increased LV mass index represented one-third of the study population with a greater percentage of the concentric hypertrophy pattern than the eccentric hypertrophy pattern (table 3 and 4).

**Table 3:** Baseline CT data of the study population

Variables	Mean ± SD
LV mass, gm	193 ± 60
LV mass index, (g/m^2^)	95.2 ± 27.5
SWT, mm	10 ± 1.9
PWT, mm	10.4 ± 1.8
LVEDD, mm	50.6 ± 5.8
Relative wall thickness	0.41 ± 0.07

Variable	Median (range)
CAD-RADS	1(0–5)

Variable	Number (%)
LV geometric pattern	
Normal pattern	111 (37.2%)
Concentric remodeling	83 (27.8%)
Eccentric hypertrophy	39 (13.0%)
Concentric hypertrophy	65 (21.8%)

**Table 4:** CAD severity according to the Society of Cardiovascular Computed Tomography (SCCT)

Variable	Number (%)
CAD-RADS 0	148 (49.7%)
CAD-RADS 1	36 (12.1%)
CAD-RADS 2	50 (16.8%)
CAD-RADS 3	10 (3.4%)
CAD-RADS 4	33 (11.1%)
CAD-RADS 5	21 (7%)

### Comparison analysis

**Fig. 3:**
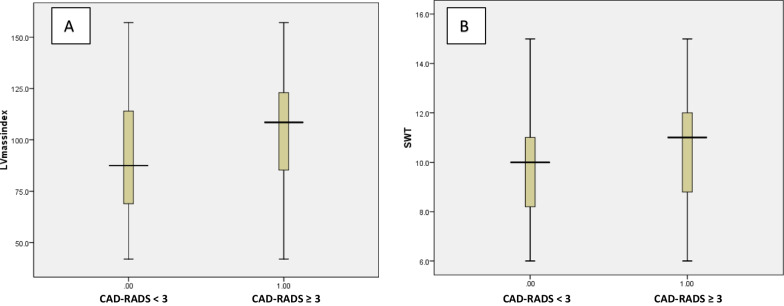
Comparison between patients with CAD-RADS < 3 vs. those with CAD-RADS ≥ 3 regarding LV mass index (panel **A**) and septal wall thickness (panel **B**)

**Fig. 4:**
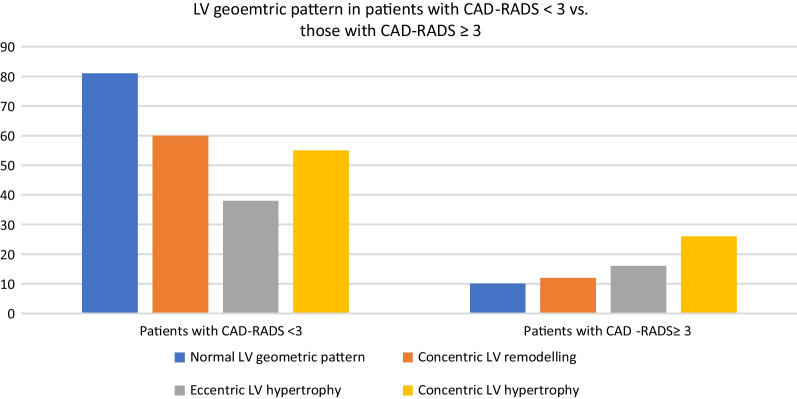
Comparison of the LV geometric pattern between patients with CAD-RADS < 3 vs. those with CAD-RADS ≥ 3

According to the result of the MSCT coronary angiography, the patients were classified into a group with less than 50% luminal diameter stenosis (CAD- RADS 0,1 and 2) and a group with 50% or more luminal diameter stenosis (CAD- RADS 3,4 and 5). A comparison between both groups was conducted (Table 5). Patients with high CAD-RADS showed higher LV mass, LV mass index, and septal wall thickness, with all three being statistically significant (Fig. 3). Patients with high CAD-RADS showed a greater percentage of concentric and eccentric hypertrophy while patients with lower CAD-RADS showed a greater percentage of normal and concentric remodeling patterns (Fig. 4).

**Table 5:** Comparison between patients with CAD-RADS < 3 and those with CAD-RADS ≥ 3

	Patients with CAD-RADS < 3 (n = 234)	Patients with CAD-RADS ≥ 3 (n = 64)	*P* value
	Mean ± SD	Mean ± SD	
Age	56.7 ± 10.5	59.4 ± 10.3	0.07
SBP, mm Hg	147.7 ± 17.0	149 ± 18.8	0.36
DBP, mm Hg	84.2 ± 8.9	85.7 ± 9.2	0.24
HR. bpm	66.9 ± 6.7	67.3 ± 7.0	0.63
Height (cm)	171.3 ± 8.2	170.6 ± 7.2	0.53
Weight(kg)	87.7 ± 16.5	87.0 ± 12.4	0.75
BMI (cm^2^/kg)	29.9 ± 5.3	30.0 ± 4.7	0.81
BSA(m^2^)	2.0 ± 0.2	2.0 ± 0.15	0.76
LV mass, gm	188.2 ± 59.1	211 ± 60.3	0.007
LV mass index, (g/m^2^)	92.9 ± 27.0	103.7 ± 27.7	0.005
SWT, mm	10.3 ± 1.8	10.9 ± 1.8	0.02
PWT, mm	9.8 ± 1.9	10.3 ± 2.0	0.07
LVEDD, mm	50.3 ± 5.6	51.8 ± 66	0.06
Relative wall thickness	0.41 ± 0.07	0.42 ± 0.08	0.33

	Number (%)	Number (%)	
Gender, male	182 (77.8%)	46 (71.9%)	0.32
LV geometric pattern			
Normal pattern	81 (34.6%)	10 (15.6%)	
Concentric remodeling	60 (25.6%)	12 (18.8%)	0.002
Eccentric hypertrophy	38 (16.2%)	16 (25%)	
Concentric hypertrophy	55 (3.5%)	26 (40%)	

*CAD-RADS score* coronary artery disease reporting and data system, *SBP* systolic blood pressure, *DBP* diastolic blood pressure

**Fig. 5:**
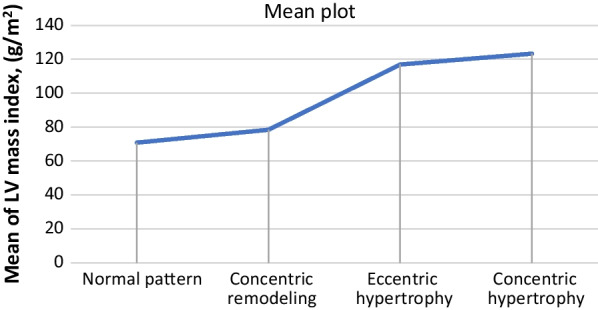
Comparison of the four LV geometric patterns regarding the LV mass index

A one-way ANOVA was conducted to compare LV mass index across the different LV geometric patterns (Fig. 5). It showed statistically significant differences between the four groups; normal, concentric remodeling, eccentric hypertrophy, and concentric hypertrophy (Table 6). Of note, post hoc analysis using Bonferroni showed no significant difference in the LV mass index between concentric hypertrophy and eccentric hypertrophy. On the contrary, there was a significant difference between all the other groups (Table 7).

**Table 6:** Comparison between the four LV geometric pattern regarding the LV mass index

	Normal pattern	Concentric remodeling	Eccentric hypertrophy	Concentric hypertrophy	*P*-value
Mean of LV mass index, (g/m^2^)	70.8	78.3	116.9	123.3	< 0.001

**Table 7:** Comparison in-between the different LV geometric patterns regarding the LV mass index

Post hoc test using Bonferroni	P-value
Normal pattern and concentric remodeling	**0.005**
Normal pattern and eccentric hypertrophy	**< 0.001**
Normal pattern and concentric remodeling	**< 0.001**
Concentric remodeling and eccentric hypertrophy	**< 0.001**
Concentric remodeling and concentric hypertrophy	**< 0.001**
Eccentric hypertrophy and concentric hypertrophy	0.07

### Correlation analysis

**Fig. 6:**
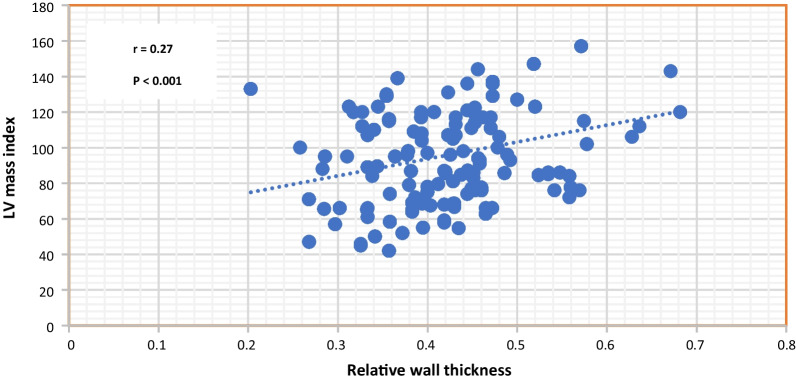
Correlation between LV mass index and relative wall thickness

There is a statistically significant weak correlation between relative wall thickness and LV mass index. This finding raises the importance of LV geometric patterns beyond the LV mass index in our analysis (Fig. 6).

### Regression analysis

**Fig. 7:**
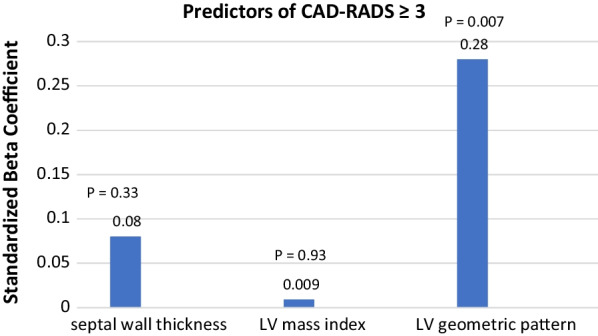
Regression analysis to predict significant CAD, CAD-RADS ≥ 3

Stepwise multiple linear regression analysis was conducted to predict the severity of coronary artery disease (Table 8). Among septal wall thickness, LV mass index, and LV geometric, the LV geometric pattern was the only independent predictor of the higher CAD-RADS (R = 0.22. adjusted R square = 0.05, *P* = 0.002) (Fig. 7).

**Table 8:** Linear Regression Analysis to predict CAD-RADS ≥ 3

Variables	R = 0.22, Adjusted R Square = 0.05, *P* = 0.002			*P*-value
	Unstandardized coefficients		Standardized coefficients	
	B	SE	Beta	
Septal wall thickness	− 0.017	0.018	− 0.083	0.33
LV mass index	0.000	0.002	− 0.009	0.93
LV geometric pattern	0.097	0.036	0.280	**0.007**

Dependent Variable: CAD-RADS ≥ 3

**Fig. 8:**
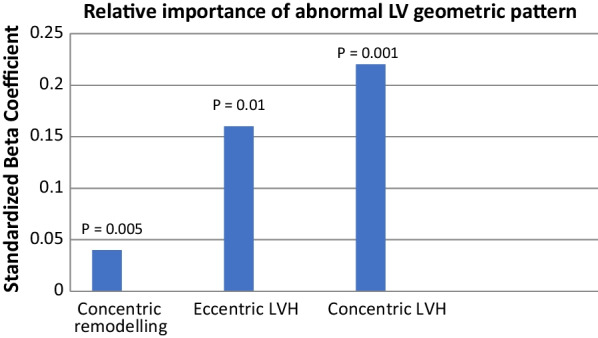
Regression analysis to show the relative importance of the different geometric patterns

Conducting multiple linear regression analysis (Table 9) had shown that LV geometric patterns associated with high RADS ordered from the highest to the lowest, were concentric LVH, Eccentric LV, and concentric remodeling (Fig. 8).

**Table 9:** Linear Regression Analysis of the abnormal LV geometric patterns to predict CAD-RADS ≥ 3

Variables	R = 0.22, Adjusted R Square = 0.04, *P* = 0.003			*P*-value
	Unstandardized coefficients		Standardized coefficients	
	B	SE	Beta	
Concentric remodeling	0.03	0.06	0.03	0.005
Eccentric LVH	0.17	0.16	0.16	0.01
Concentric LVH	0.2	0.06	0.22	0.001

Dependent Variable: CAD-RADS ≥ 3

## Discussion

This is the first study to investigate the relationship between CAD using CAD-RADS and both LV mass and its geometric patterns in hypertensive patients using a 320-detector-row CT scanner.

Hypertension consistently shows high prevalence in all countries irrespective of their socio-economic status. The disability-adjusted life years due to hypertension has increased by 40% since 1990. Ischemic heart disease stands as the most common cause of systolic blood pressure-related deaths per year [1–3].

Coronary computed tomography angiography (CCTA) is a widely available, clinically validated, noninvasive imaging tool that is used to evaluate the coronary arterial tree. CAD-RADS is a standardized system for reporting the CCTA results. It has the advantages of improving communication with the referring physicians and facilitating research work, resulting in better patient care and outcomes [22]. CCTA using the wide-detector CT scanner (320-detector-row CT scanner), besides assessing the coronary arteries, has also allowed for obtaining accurate volumetric data of the cardiac chambers, especially the left ventricle (LV) [26, 27].

Left ventricular hypertrophy determined by echocardiography is an independent risk factor for increased cardiovascular morbidity and mortality. This well-established relationship is continuous and graded [7–11, 28, 29].

Koren et al. and Muiesan et al. conducted two prospective studies including relatively healthy adults with essential hypertension, following them up for ten years. Levy et al. also conducted a prospective study that included Framingham study participants without prevalent cardiovascular disease with four years of follow-up. The three studies showed an adverse prognostic value of LVH determined by echocardiography using different cardiovascular disease endpoints, but all included MI [7, 10, 30].

Tang et al. conducted a non-randomized study on the members of the National Heart, Lung, and Blood Institute Family Heart Study and the Hypertension Genetic Epidemiologic Network study. They found a significant association between coronary artery calcium score (CAC) and LV mass index. This relationship was stronger in African Americans than whites [31].

Altunkan et al. conducted a study on two hundred forty-nine asymptomatic hypertensive patients. They found a direct relationship between LVH and coronary artery calcium where patients who were CAC positive showed significantly greater LVM than those who were CAC negative [32].

Mintu et al. conducted an observational study on one thousand and sixteen patients with coronary heart disease and a follow up period of three and half years. They found that LV mass index adjusted for clinical and other echocardiographic parameters was significantly associated with both all-cause mortality and sudden or arrhythmic death. This association maintained its significance even in patients with normal LV systolic function (defined as more than 55%) [33].

In agreement with the previously mentioned studies, our study using a 320-detector-row CT scanner showed that LV mass, LV mass index, and septal wall thickness were significantly higher in patients with significant coronary artery disease (CAD-RAD ≥ 3) than in those with non-obstructive CAD (CAD-RADS < 3). This finding confirms the significant relationship between LVH and CAD with the use of a wide detector CT scanner.

Despite representing different hemodynamic loads with different pathophysiologic mechanisms, the body of evidence that supports the prognostic value of the LV geometric pattern beyond LV mass is small, less clear, and sometimes contradictory [10, 14, 27, 34, 35].

Koren et al. first reported the prognostic value of the LV geometric pattern in patients with uncomplicated essential hypertension. The mortality rate over a follow-up period of 10 years was highest in patients with concentric LVH, followed by patients with eccentric hypertrophy and concentric remodeling (24%, 10%, and 6% respectively) [10].

Verdecchia et al. confirmed the adverse prognosis of concentric remodeling in 694 hypertensive patients, even after adjustment for several covariates (age, gender, diabetes, left ventricular mass index, and blood pressure) [12]. One year later, Verdecchia et al. published a study on hypertensive patients with LVH, where he couldn't confirm the prognostic value for the LV geometric pattern beyond that of the LV mass [14].

In the Framingham Heart Study, three thousand two hundred and sixteen Caucasian population sample had been investigated and followed up for 8 years. The outcome events were incident cardiovascular disease and death. The event rates were greatest in the concentric LVH group and lowest in the group with normal geometric pattern in both genders. However, this association eroded after adjustment for the LV mass [13].

The LIFE echo sub-study investigated nine hundred and sixty-three patients with a mean age of 66 years. One hundred and forty-nine patients had CAD and eight hundred and fourteen did not. The definition of CAD in this study was clinical, history of angina pectoris, myocardial infarction, or electrocardiographic evidence of myocardial infarction. This definition may underestimate the true prevalence of CAD. This study found that the prevalence of eccentric hypertrophy was significantly higher in patients with CAD [35].

Ghali et al. investigated nine hundred and eighty-eight predominantly African American hypertensive patients. The definition of CAD was the presence of at least one vessel with seventy percent diameter narrowing or more by conventional coronary angiography in multiple projections. They were followed up for a mean of nine years. They found that all-cause mortality increased progressively with different LV geometric patterns (normal pattern, concentric remodeling, eccentric hypertrophy, and concentric hypertrophy, sorted in ascending order). Cardiac deaths increased progressively from eccentric to concentric hypertrophy. The adjusted relative risk and 95% confidence of interval of cardiac death were significantly increased in patients with concentric hypertrophy (RR 2.21 to 297 for patients with and without CAD respectively). Patients with eccentric hypertrophy showed a moderate increase in cardiac death (RR 1.3 to 2.87 for patients with and without CAD respectively). Of note, concentric remodeling was not associated with an increase in the relative risk of cardiac death (RR 0.98 and 0.73 for those with or without CAD, respectively) [34].

Altunkan et al. found that significantly more patients with LV concentric hypertrophy had a positive CAC (57% of the patients in this group) compared to other LV geometric patterns. There was no significant difference in the number of CAC-positive patients among the other groups (eccentric hypertrophy, concentric remodeling, and normal group). In addition, the patient in the concentric hypertrophy group shows an average coronary calcium score (315 ± 761) that is significantly higher than other groups [32].

Uçar et al. studied two hundred and fifty hypertensive patients who underwent invasive coronary angiography. Coronary angiography was performed to investigate suspected ischemic heart disease based on clinical indications. The cumulative SYNTAX score (SS) was calculated to show the complexity and extent of CAD. They found that patients with concentric hypertrophy show the highest SS compared to other groups with a *P*-value of less than 0.05. Patients with eccentric hypertrophy show significantly higher SS than patients with concentric remodeling and normal geometry. Patients with concentric remodeling and normal geometry showed similar SS. Of note, concentric LV hypertrophy was the most important independent predictor of SS by regression analysis [37].

The previously mentioned studies investigated different types of populations with different study designs. The outcomes were all-cause mortality, cardiovascular mortality, or coronary artery disease. The extent of CAD was used as a surrogate for increased cardiovascular mortality. In the previous studies, multiple methods were used for CAD assessment; clinical, non-invasive (stress ECG37, nuclear imaging33, or electron beam CT to measure CAC), or invasive coronary angiography. There were also limitations regarding the methodology, the small number of patients in each LV geometric pattern, and the low? number of outcome events.

In our study, patients with high CAD-RADS showed a significantly higher percentage of abnormal LV geometric patterns (concentric and eccentric hypertrophy represent 65% of this group) while patients with low CAD-RADS showed a higher percentage of normal and concentric remodeling patterns (about 60% of this group). Patients with normal LV geometry is the predominant pattern in patients with low CAD-RADS (about one-third) and is the least predominant pattern (about 15%) in those with high CAD-RADS.

Among septal wall thickness, LV mass index, and LV geometry, LV geometric pattern was the only independent predictor of the high CAD-RADS (R = 0.22. adjusted R square = 0.05, *P* = 0.002). LV concentric hypertrophy is the most important predictor of high CAD-RADS (standardized co-efficient of 0.22, *P* = 0.001) followed by eccentric hypertrophy (standardized co-efficient of 0.16, *P* = 0.01) and lastly concentric remodeling (standardized co-efficient of 0.22, *P* = 0.001). There was no significant difference in the LV mass index between the LV concentric hypertrophy group and the LV eccentric hypertrophy group which confirms the independent role of LV concentric hypertrophy in predicting high CAD-RADS. The weak correlation between LV mass index and relative wall thickness emphasizes the independent adverse prognostic value of concentric remodeling in patients with lower LV mass index (normal pattern and concentric remodeling).

The LV geometric pattern in hypertensive patients shows a graded association with CAD: Concentric LV hypertrophy shows the highest cardiovascular risk, followed by eccentric hypertrophy and then concentric remodeling. The normal LV geometric pattern is associated with the lowest cardiovascular risk. Therefore, hypertension should be viewed as a disease with different subgroups, each heralding a different prognosis.

## Limitation

- Cardiac CT, despite being more accurate than echocardiography (overcoming the problems of operator dependency, poor window, and the assumption of a uniform LV wall thickness in two-dimensional echocardiography) cannot be used for routine assessment of the LV mass due to the risk of radiation, cost, and availability
- The effect of patient gender could not be addressed in our study.
- Dividing the patients into four LV geometric patterns and separating them based on CAD-RADS led to a small number in each group. Therefore, a larger number of patients in each group is needed to strengthen our results.

## Conclusions

Abnormal LV geometric pattern (concentric and eccentric hypertrophy) is associated with high CAD-RADS. Among septal wall thickness, LV mass index, and LV geometry, the LV geometric pattern is the only independent predictor of high CAD-RADS. LV concentric hypertrophy is the most important predictor of high CAD-RADS, followed by eccentric hypertrophy then concentric remodeling. Therefore, assessment of the LV geometric pattern with the use of a wide scanner CT can improve the risk stratification of patients with hypertension.

## Data Availability

The datasets used and/or analysed during the current study are available from the corresponding author on reasonable request.

## Abbreviations

ANOVA: Analysis of variance
BMI: Body mass index
BSA: Body surface area
CAC: Coronary artery calcium
CAD: Coronary artery disease
CAD-RADS: Coronary artery disease reporting and data system
CT: Computed tomography
ECG: Electrocardiogram
FOV: Field of view
g: Gram
Kv: Kilo volt
LV: Left ventricle
LVID: Left LV end-diastolic inner dimension
LVH: Left ventricular hypertrophy
mA: Milliampere
m: Meter
MSCT: Multi-slice computed tomography
MPR: Multi-planar reformatted
SWT: Septal wall thickness
RWT: Relative wall thickness
SCCT: Society of cardiovascular computed tomography
SD: Standard deviation
SPSS: Statistical Package of Social Science
SWT: Septal wall thickness
SS: SYNTAX score
WHO: World health organization
